# Treatment-as-prevention: stopping the epidemic of HIV

**DOI:** 10.1186/1758-2652-13-S4-O13

**Published:** 2010-11-08

**Authors:** B Williams

**Affiliations:** 1 1218 Le Grand Saconnex, Geneva, Switzerland

## 

Anti-retroviral drugs an reduce plasma HIV viral load by 3 to 4 orders of magnitude raising the possibility that treatment can be used not only to save individual lives but also to stop transmission. Modelling studies suggest that with the effective use of ART transmission of HIV could be eliminated within ten years and HIV infection within 40 years. The ides of using treatment as prevention has generated considerable interest and a certain amount of controversy. This presentation will describe the potential impact of *treatment-as-prevention*, compare the results with treatment-as-prophylaxis, and consider some of the key factors that could compromise the impact of this approach. These include coverage, compliance, the extent of viral load suppression, the reduction in transmission, the likelihood of virological failure, viral rebound and drug resistance. Possible ways of increasing the impact through targeting of the intervention will be discussed. Finally, some consideration will be given as to how best to test and possibly implement a programme of using treatment as prevention.

